# Supplementation with xylanase and β-xylosidase to reduce xylo-oligomer and xylan inhibition of enzymatic hydrolysis of cellulose and pretreated corn stover

**DOI:** 10.1186/1754-6834-4-18

**Published:** 2011-06-24

**Authors:** Qing Qing, Charles E Wyman

**Affiliations:** 1 Center for Environmental Research and Technology, Bourns College of Engineering, University of California, Riverside, 1084 Columbia Avenue, Riverside, California 92507, USA; 2 Chemical and Environmental Engineering Department, Bourns College of Engineering, University of California, Riverside, 1084 Columbia Avenue, Riverside, California 92507, USA

## Abstract

**Background:**

Hemicellulose is often credited with being one of the important physical barriers to enzymatic hydrolysis of cellulose, and acts by blocking enzyme access to the cellulose surface. In addition, our recent research has suggested that hemicelluloses, particularly in the form of xylan and its oligomers, can more strongly inhibit cellulase activity than do glucose and cellobiose. Removal of hemicelluloses or elimination of their negative effects can therefore become especially pivotal to achieving higher cellulose conversion with lower enzyme doses.

**Results:**

In this study, cellulase was supplemented with xylanase and β-xylosidase to boost conversion of both cellulose and hemicellulose in pretreated biomass through conversion of xylan and xylo-oligomers to the less inhibitory xylose. Although addition of xylanase and β-xylosidase did not necessarily enhance Avicel hydrolysis, glucan conversions increased by 27% and 8% for corn stover pretreated with ammonia fiber expansion (AFEX) and dilute acid, respectively. In addition, adding hemicellulase several hours before adding cellulase was more beneficial than later addition, possibly as a result of a higher adsorption affinity of cellulase and xylanase to xylan than glucan.

**Conclusions:**

This key finding elucidates a possible mechanism for cellulase inhibition by xylan and xylo-oligomers and emphasizes the need to optimize the enzyme formulation for each pretreated substrate. More research is needed to identify advanced enzyme systems designed to hydrolyze different substrates with maximum overall enzyme efficacy.

## Background

The abundance and diversity of feedstocks and the potential to prevent changes of the thermal equilibrium of the atmosphere caused mostly by carbon dioxide makes conversion of cellulosic biomass to ethanol and other fuels more advantageous than fossil fuels or first-generation corn ethanol [1]. However, the inherent recalcitrance of cellulosic materials means that they require more severe processing than do corn or sugarcane. In addition, even for biomass pretreated under optimum conditions by leading pretreatment technologies, very high enzyme doses are still required to achieve high-yield conversion of polymeric cellulose and hemicellulose into sugar monomers that can be utilized by fermentative microorganisms [2]. Thus, the costs of enzymes and pretreatment are the major barriers to low costs by biological processing of cellulosic biomass, and must be lowered substantially to make the cost of cellulosic ethanol competitive with that of fossil fuels or corn ethanol [3]. Advances in current pretreatment technologies to generate more digestible substrates and modifying hydrolytic enzyme cocktails to improve enzyme efficacy could benefit from more in-depth and comprehensive understanding of the interaction between pretreated biomass and enzymes, and the synergism operating between different enzyme components in producing fermentable sugars.

Enzymatic digestion of cellulosic materials involves synergic action of a group of different functional enzymes [4,5]. In general, endoglucanases (EC 3.2.1.4) and exoglucanases (cellobiohydrolases; CBHs) break down cellulose at the solid-liquid interface, whereas accessory enzymes such as hemicellulases, acetyl xylan esterase [6], α-L-arabinofuranosidase, feruloyl esterase and p-coumaroyl esterase help cleave physical shields that cover cellulose microfibrils [7,8]. Therefore, the accessibility of the cellulose surface to cellulases and the subsequent efficacy of these enzymes have been identified as important factors that dominate cellulose hydrolysis yield [9-12]. However, the heterogeneous nature of cellulosic materials makes enzyme access to the cellulose surface very difficult, and the presence of lignin and hemicelluloses in pretreated biomass have been suggested to cause major obstacles to enzymatic digestion of cellulosic materials, by physically blocking the access of cellulase and non-productively binding with enzymes [13-17]. Our recent research suggests that xylose, and particularly soluble xylo-oligomers released from hemicelluloses during enzymatic hydrolysis, could present an additional important barrier to enzyme action by competitively inhibiting cellulase activity [18]. Furthermore, it is difficult to totally hydrolyze these xylo-oligomers and reduce their effect within a typical hydrolysis span of 72 hours using moderate doses of commonly used and commercially available enzymes such as cellulase preparations (Spezyme CP) and β-glucosidase (Novozyme 188). Thus, there is a need to understand the mechanisms of cellulase inhibition by solid xylan and soluble xylo-oligomers and reduce their negative effects on cellulose hydrolysis.

In this study, xylanase (Multifect; Genencor) and/or β-xylosidase were added to cellulase to evaluate the synergism of hemicellulases and cellulases in hydrolyzing pure cellulose and the cellulose in pretreated biomass. For comparison, corn-stover solids were used, with two different pretreatments: 1) ammonia fiber expansion (AFEX), which allows the stover to retain almost all of the xylan and other hemicellulose, cellulose and lignin fractions, and 2) dilute acid, which results in a relatively low amount of xylan left in the solid residue. Because enzymes must adsorb to the solid substrate surface before the hydrolyzing reaction occurs, adsorption of cellulase and hemicellulase on different substrates was measured to understand the interactions of the different enzymes and the substrates. In addition, the effects of xylanase and β-xylosidase in reducing inhibition by xylo-oligomers were evaluated, and different cellulase and hemicellulase supplementation sequences were used to test our hypothesis that hydrolyzing xylans and xylo-oligomers before adding cellulase could reduce loss of enzyme activity.

## Methods

### Materials

Microcrystalline cellulose (Avicel PH-101, cellulose content > 97%, lot number 1300045 32806P01) and birchwood xylan (lot number 038K0751) with a xylan content measured at ~85% [19] were purchased from Sigma-Aldrich (St. Louis, MO, USA). Corn stover was provided by the National Renewable Energy Laboratory (NREL, Golden, CO, USA) from a lot obtained from the nearby Kramer Farm (Wray, CO, USA). The AFEX-pretreated corn stover (pretreatment condition is shown in Table 1) was generously provided by the Bruce Dale group at Michigan State University. The AFEX pretreatment conditions were 1:1 ammonia to biomass loading, 60% moisture (dry weight basis), treated at 140°C for 15 minutes residence time. The pretreated samples were kept in the hood to remove residual ammonia and stored at 4°C until further use.

**Table 1:** Compostion of corn stover and washed solids from its pretreatment by dilute acid and AFEX technologies.

		**Yield, %**^**1**^		
**Substrates**	**Pretreatment conditions**	**Glucan**	**Xylan**	**Lignin**

Untreated corn stover	NA^2^	39.1 ± 0.4	23.7 ± 0.3	19.3 ± 0.5

Dilute acid-pretreated	140°C, 1% sulfuric acid, 40 minutes	57.6 ± 0.2	5.7 ± 0.7	22.1 ± 0.4

AFEX^**3**^-pretreated	90°C, 220 psi, 1:1 NH_3 _to Biomass, 5 minutes	39.6 ± 0.5	24.5 ± 0.4	18.2 ± 0.9

^1^Data are mean ± SD.

^2^Not applicable.

^3^Ammonia fiber expansion.

Cellulase (Spezyme^® ^CP, lot number 301-05330-205, cellulase activity 59 ± 5 filter paper units (FPU)/ml, xylanase activity 2622 OSX (oat spelt xylan)/ml, protein content 123 ± 10 mg protein/ml), xylanase (Multifect^®^, lot number 301-04021-015; xylanase activity 25203 OSX/ml, protein content 42 ± 5 mg protein/ml) and β-xylosidase (lot number 20050881-0882, protein content 75 ± 5 mg protein/ml) enzyme preparations were all supplied by Genencor (a Danisco Division, Rochester, NY, USA). The β-glucosidase (Novozyme 188; 18066K0676, 665 CBU/ml, protein content 140 ± 5 mg protein/ml) was purchased from Sigma-Aldrich. The β-xylosidase preparation used in this study was a non-commercial enzyme expressed in a *Trichoderma *strain in which the major cellulase genes were deleted, specifically CBH1, CBH2, EG1 and EG2.

### Dilute acid pretreatment

The dilute acid-pretreated corn stover used in this study was pretreated at 140°C for 40 minutes with 1% by weight (wt%) sulfuric acid in a 1 litre stainless-steel batch reactor from Parr instruments (Moline, IL, USA) with a solids loading of 5 wt%, these conditions having been previously determined to maximize recovery of total glucose plus xylose [20]. The reactor vessel was sealed and placed in a sand bath (model SBL-2D; Techne Co., Princeton, NJ, USA) set at 320°C for fast heating, and transferred to a sand bath at 140°C to maintain the target temperature. Mechanical agitation at 200 rpm by a turbine impeller was applied during heating, reaction and cooling. After 40 minutes, the reactor was quenched quickly in iced water to room temperature, and after the solution cooled down to room temperature, the solids were separated from the liquid hydrolyzate by centrifugation (model 5424; Eppendorf, Hauppauge, NY) at 18895 g for 5 minutes. The solids were collected and washed at least three times with 1 litre deionized water to neutralize and remove possible degradation products.

### Xylo-oligomer production

A xylo-oligomer rich solution was produced by pretreatment of birchwood xylan (lot number 038K0751, Sigma Chemicals) with water only at 200°C for 15 minutes at 10 wt% solids loading in the same 1 litre stainless-steel Parr reactor and using the same procedure described above. After separation of the liquid hydrolyzate from the solid residue, the liquid portion was collected for further use and stored at 50°C in an incubation shaker (Infors HT, Bottmingen, Switzerland) to prevent precipitation of high degree of polymerization (DP) xylo-oligomers [21]. To avoid possible degradation or precipitation of xylo-oligomers, this solution was usually generated the day before use.

### Sugar quantification

All monomeric sugars were quantified by high-performance liquid chromatography (HPLC) (Waters Alliance HPLC System, model 2695; Waters Corporation, Milford, MA, USA) with an Aminex column (HPX-87P; Bio-Rad Laboratories, Hercules, CA, USA) and a refractive index detector (2414; Waters). Liquid samples were neutralized when appropriate and filtered through 0.2 μm nylon filter vials (Alltech Associates Inc., Deerfield, IL, USA), transferred by pipette into 500 μl polyethylene HPLC vials (Alltech Associates Inc.), and kept refrigerated at 4°C until analyzed. A series of sugar standards with different concentrations were run together with the liquid samples, and used as the calibration basis.

### Compositional analysis

To determine the structural carbohydrates and lignin in raw and pretreated corn stover, all samples were dried to constant weight by placing in a convection oven (series 0504-6593; Barnstead Lab-Line, Melrose Park, IL, USA) at temperature of less than 45°C. These constituents were measured using a two-step acid hydrolysis method to fractionate the biomass into forms that are more easily quantified. The acid-insoluble lignin including ash was quantified by gravimetric analysis. During acid hydrolysis, the polymeric carbohydrates were hydrolyzed into monomers that are soluble in the hydrolysis liquid and could be measured by HPLC. A series of sugar-recovery standards were run in parallel to correct for sugar degradation during this process [19].

### Enzymatic hydrolysis

All enzymatic hydrolysis experiments were performed according to the National Renewable Energy Laboratory analytical procedures at 2% (w/v) solids loading with 0.05 mol/l citrate buffer (pH 4.8) in a thermostatically controlled shaker at 50°C [22]. To prevent possible microbial growth on the sugars generated, 100 μL of 2% sodium azide was added before enzymes. Spezyme CP cellulase (16.1 mg protein/g glucan), Novozyme 188 β-glucosidase (3.16 mg protein/g glucan), Multifect xylanase (16.1 mg protein/g glucan) and the non-commercial β-xylosidase (32.2 mg protein/g glucan) were added to the hydrolysis broth with different loadings and in different combinations. Hydrolysis samples taken after 4, 24, 48 and 72 hours of hydrolysis were analyzed according to the sugar-quantification method described above to follow the reaction course and determine final yields.

### Protein adsorption

Protein-adsorption experiments were carried out at 4°C to prevent hydrolysis of the substrate at a concentration of 1% solids in a total volume of 1.1 ml in 1.5 ml microcentrifuge tubes (Lobind; Eppendorf) (protein loss <3%). Cellulase, β-glucosidase, xylanase and β-xylosidase were added separately to bring the final protein concentration to a range from 0 to 15 mg/ml. The samples were then rotated slowly at 4°C in a refrigerator for 4 hours to allow equilibration, followed by separation in a centrifuge (model 5415D; Eppendorf) at 4°C at a maximum speed of 2897968 g for a minimum of 10 minutes. The resulting solids were dried in a convection oven at 105°C overnight. Following a protocol developed previously, the protein adsorbed on solids was directly determined by measuring the nitrogen content of the samples using an elemental analyzer (Flash 1112 CHNOS Analyzer, CE Elantech, Lakewood, NJ, USA). For nitrogen-content analysis, the samples were weighed (~ 6 mg) into a tin capsule, which was then sealed, and were run on the analyzer along with appropriate nitrogen standards such as aspartic acid or BBOT (2,5-Bis-(5-tert.-butyl-benzoxazol-2-yl)-thiophen) [23]. Protein adsorption could then be calculated based on a mass balance for nitrogen, with a nitrogen factor (NF) of 8.40 for Spezyme CP cellulase, 8.28 for Multifect xylanase and 3.25 for Novozyme 188 β-glucosidase [23,24]. The NF for Genencor β-xylosidase was determined to be 7.85 ± 0.3 in this study, using the same method described by Kumar and Wyman [23]

The Langmuir isotherm equation [25] was applied to describe adsorption by the following expression, with the parameters estimated by non-linear regression using Polymath software (Polymath Software, P. O. Box 523 Willimantic, CT 06226-0523 USA):

where E_bound _and E_free _represent the amount of enzyme adsorbed to the solids (mg/g substrate) and the enzyme remaining in the solution (mg/ml), respectively; S is the substrate concentration in mg/ml; σ is the maximum adsorption capacity in mg/g substrate; and *K*_d _(L/g) is the equilibrium constant.

### Enzyme-supplementation sequence

To elucidate the importance of removing or totally hydrolyzing xylo-oligomers on cellulose hydrolysis, hemicellulases (xylanase and β-xylosidase) were added several hours before or after addition of cellulase. To eliminate the effects of lignin, pure Avicel cellulose was combined with mixed DP xylo-oligomers at a weight ratio of 8:5 cellulose to the equivalent amount of xylose in the xylo-oligomers, to simulate the glucan to xylan ratio for solids produced by AFEX pretreatment. One part of this mixture was incubated at 50°C with xylanase and β-xylosidase only, at a loading of 30 mg protein/g equivalent xylose (xylanase: β-xylosidase 1:1) for 2, 4, 24 and 72 hours. After incubation, cellulase was added at a loading of 10.7 mg/g glucan (about 5 FPU/g glucan) with β-glucosidase supplementation at a cellobiase units (CBU): FPU ratio of 2:1. As a comparison, xylanase and β-xylosidase were also added to another portion of samples that were initially incubated with cellulase and β-glucosidase for 2, 4 or 24 hours. Samples of cellulose and a mixture of cellulose and xylo-oligomers were run with cellulase and β-glucosidase only as controls. The time of the samples taken before cellulase addition was designated as time 0 even if they were incubated with hemicellulase.

## Results and Discussion

### Composition of solids after dilute acid and AFEX pretreatments

The disruption of the lignin-hemicellulose matrix enhances the susceptibility of cellulosic biomass solids to attack by enzymes. Low and neutral pH pretreatment technologies such as dilute acid for the former and liquid hot water for the latter usually remove a large fraction of the hemicellulose from biomass, but remove very little lignin. Furthermore, pretreatments at low pH hydrolyze most of the hemicellulose to monomers, whereas pretreatments at near neutral pH produce mostly oligosaccharides with some monomers. Depending upon the pretreatment severity, some degradation products are released over this range of pH conditions. By contrast, applying a high pH pretreatment, such as lime, soaking in aqueous ammonia (SAA) or ammonia recycle percolation (ARP), leaves the majority of the hemicellulose in the solids while releasing large amounts of lignin [26,27]. An anomaly at high pH is that treating cellulosic biomass with anhydrous ammonia in the AFEX method at relatively mild temperatures (60-100°C) and high pressures (250-300 psi) for short durations, followed by rapid decompression, breaks up the recalcitrant barriers to increase the exposed surface area without much apparent change in the solids composition [28,29]. Therefore, solids from dilute acid and AFEX pretreatments were chosen to explore how xylan-hydrolyzing enzymes could influence the enzymatic hydrolysis of cellulose in the presence of the lowest to highest possible xylan contents while retaining most of the lignin from the original corn-stover solids.

The glucan and xylan contents in corn- stover solids before pretreatment and after application of dilute acid and AFEX are summarized in Table 1. As expected, and shown in earlier studies, AFEX did not substantially change the carbohydrate or lignin contents, but still enabled high enzymatic conversion through the combined chemical and physical effects of lignin relocation, cellulose decrystallization and increased surface area [26,27,29]. By contrast, dilute acid pretreatment performed at 140°C with 1% sulfuric acid for 40 minutes hydrolyzed a large portion of the hemicelluloses into sugars in solution. As measured in this study, the AFEX-pretreated corn-stover solids contained 39.6% glucan and 24.5% xylan, virtually the same as in the raw corn stover, whereas the solids after dilute acid pretreatment contained 57.6% glucan and only 5.7% xylan. The lignin content for the non-pretreated feedstock and the AFEX-pretreated solids were very similar, whereas that of the dilute acid-pretreated solids was slightly higher, because removal of so much xylan overwhelmed the effect of the limited solubilization of lignin.

### The effects of xylanase and β-xylosidase on enzymatic hydrolysis of cellulose and pretreated corn stover

To overcome the negative effects of residual hemicellulose on enzymatic hydrolysis of pretreated biomass [30-34], some recent research added xylanase to cellulase as an accessory activity to reduce the physical barrier of hemicelluloses [8]. Their study also found a strong linear relationship between release of xylobiose and cellobiose, which reinforced their hypothesis that the polysaccharide network in the cell-wall matrix was strongly interconnected [8]. The substantial inhibition of cellulase by xylo-oligomers revealed by our recent research emphasized the benefits of removing or hydrolyzing xylan and xylo-oligomers in enhancing cellulose digestibility, and prompted our addition of xylanase and β-xylosidase to cellulase to determine the synergic effects of these two classes of enzymes on hydrolyisis of different substrates [18]

**Figure 1:**
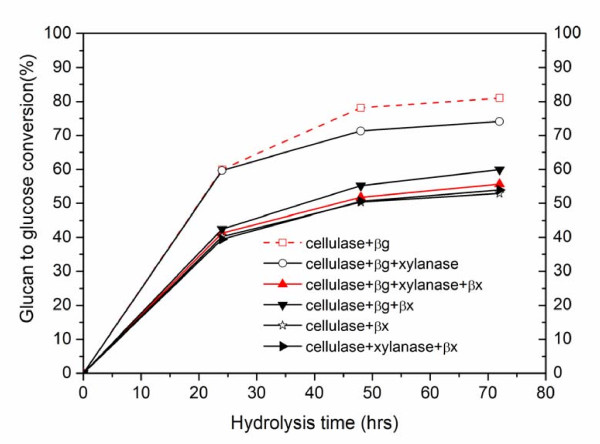
**Enzymatic hydrolysis of 2% solids loading Avicel by different enzyme combinations**. Spezyme CP cellulase loading was 16.1 mg protein/g glucan (~7.5 filter paper units (FPU)), and a ratio of 2:1 (cellobiase units (CBU): FPU) was used for β-glucosidase supplementation. Multifect xylanase was supplemented on a protein concentration basis equal to that of cellulase, and the β-xylosidase loading was 32.2 mg protein/g glucan.

The effect of xylanase and β-xylosidase addition on hydrolysis of pure cellulose is shown in Figure 1. An enzyme loading of 16.1 mg protein (~7.5 FPU/g glucan) was used for all cellulase additions, whereas β-glucosidase was supplemented at a ratio of 2:1 (CBU: FPU). The mass of xylanase protein added was equal to that for cellulase (16.1 mg protein/g glucan), but the quantity of β-xylosidase (32.2 mg protein/g glucan) added was twice that of the cellulase or xylanase to maximize removal of xylo-oligomers and especially low DP soluble xylo-oligomers from the hydrolysis broth. Unexpectedly, addition of extra xylanase or β-xylosidase reduced cellulose conversions even though small amounts of cellulase and β-glucosidase activities were detected in Multifect xylanase (Figure 1). Thus, adding 7.5 FPU plus 15 per gram glucan hydrolyzed 81% of the cellulose into monomeric glucose within 72 hrs. However, addition of xylanase dropped the glucan to glucose yield by 7%, whereas addition of β-xylosidase dropped the yield by 15%. As expected, due to low β-glucosidase activity in Spezyme CP, without adding β-glucosidase, the glucan to glucose conversion was only 53% and a large amount of cellobiose accumulated in the hydrolysis broth. This negative synergy of these enzymes may be due to hemicellulases hindering cellulase and β-glucosidase or occupation of cellulose catalytic sites non-productively [18].

**Figure 2:**
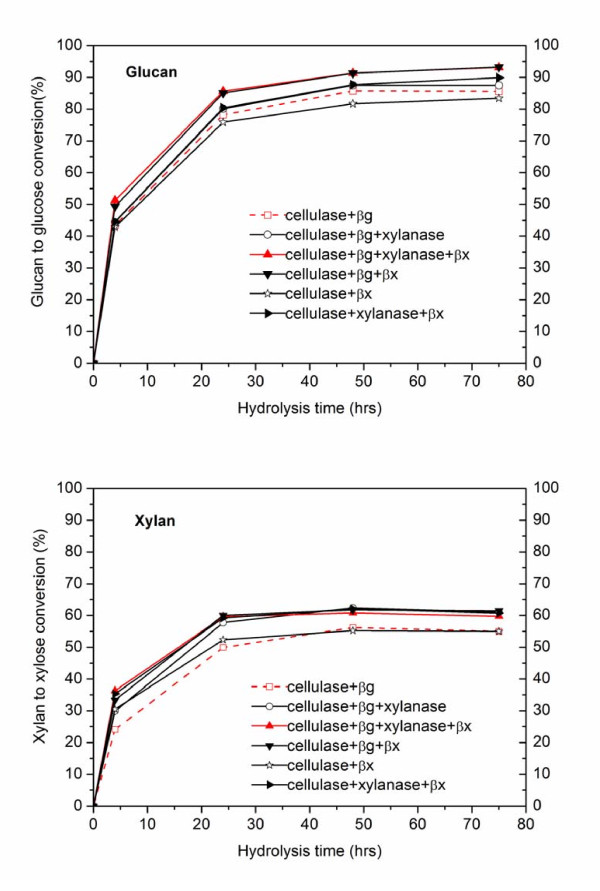
**Enzymatic hydrolysis of 2wt% loading washed dilute acid-pretreated corn-stover solids with different enzyme combinations**. Spezyme CP cellulase loading was 16.1 mg protein/g glucan (~7.5 filter paper units (FPU)) and a ratio of 2:1 (cellobiase units (CBU: FPU) was used for β-glucosidase supplementation. Multifect xylanase was added on a protein concentration basis equal to that of cellulase and the β-xylosidase loading was 32.2 mg protein/g glucan to maximize conversion of dissolved xylo-oligomers to xylose.

**Figure 3:**
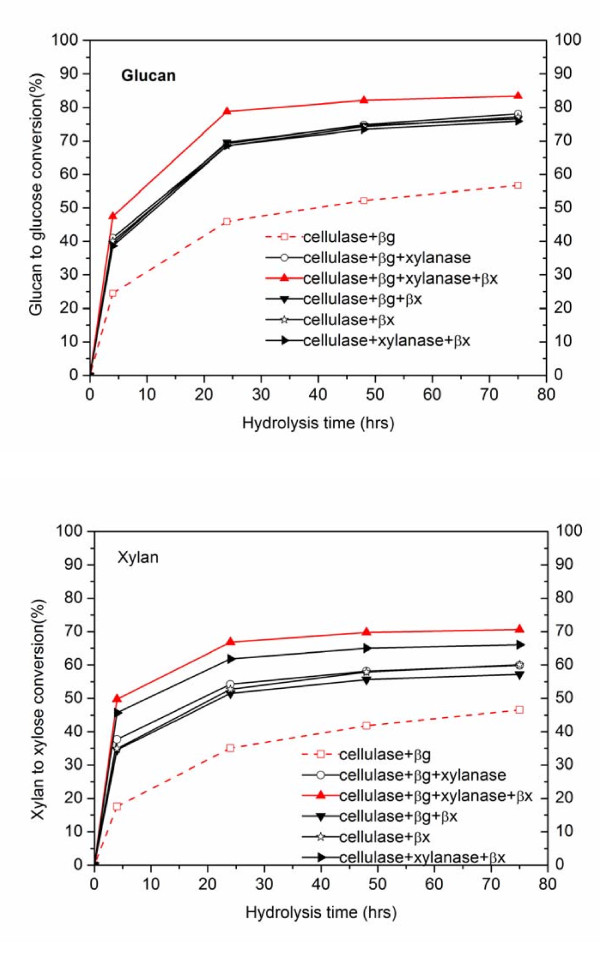
**Enzymatic hydrolysis of 2wt% loading of washed ammonia fiber expansion (AFEX)-pretreated corn-stover solids with different enzyme combinations**. Spezyme CP cellulase loading was 16.1 mg protein/g glucan (~7.5 filter paper units (FPU)) and a ratio of 2:1 (cellobiase units (CBU: FPU) was used for β-glucosidase supplementation. Multifect xylanase was added on a protein concentration basis equal to that of cellulase and the β-xylosidase loading was 32.2 mg protein/g glucan to maximize conversion of dissolved xylo-oligomers to xylose.

In sharp contrast to the behavior shown in Figure 1 with pure cellulose, adding xylanase or β-xylosidase improved both glucose and xylose yields for enzymatic hydrolysis of biomass pretreated by either dilute acid or AFEX (Figure 2 and 3). For the runs with dilute acid-pretreated corn stover shown in Figure 2A and 2B, addition of xylanase and β-xylosidase or just the latter enhanced glucose yields by 8% and xylose yields by 5% compared with hydrolysis with just cellulase and β-glucosidase. For AFEX-pretreated corn stover with nearly complete retention of xylan in the solids, addition of either xylanase or β-xylosidase increased both glucose and xylose yields significantly, as shown in Figure 3A and 3B. In particular all four enzyme preparations resulted in the highest glucan to glucose conversions of 83%, 26% > when just cellulase and β-glucosidase were used and xylan to xylose conversion was enhanced by 24% with xylanase and β-xylosidase (Figure 3B), as expected. It was interesting to note that supplementation of cellulase with β-xylosidase resulted in better glucose and xylose yields than adding β-glucosidase (Figure 3A, 3B). Furthermore, xylanase and β-xylosidase supplementation was more beneficial to the AFEX substrate that was richer in xylan and the enhanced glucose and xylose yields suggest a relationship between xylan or xylo-oligomer removal (or hydrolysis) and glucan to glucose conversion. Together with the observations shown in Figure 1, these results elucidate that the extra xylanase and β-xylosidase protein significantly hydrolyzed inhibitory xylo-oligomers into xylose and hence results into higher cellulase efficacy but do not necessarily improve cellulose conversion to glucose. However, advanced enzyme formulations are needed to reduce the negative effects of xylo-oligomers and xylan whereas reducing the total protein mass needed to achieve a given yield.

### Protein adsorption to different substrates

**Figure 4:**
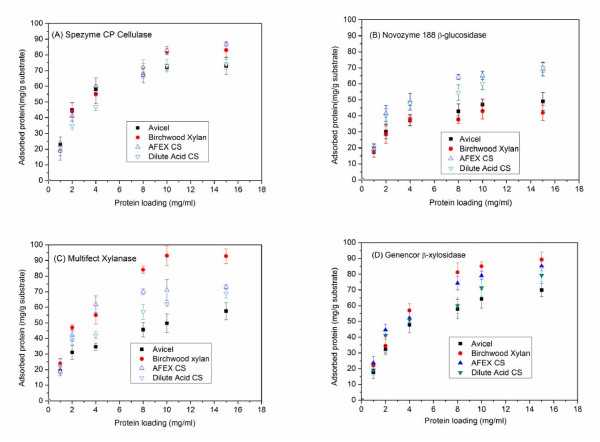
**Protein-adsorption profiles. (A) **Spezyme CP cellulase, **(B) **Novozyme 188 β-glucosidase, **(C) **Multifect xylanase and **(D) **Genencor β-xylosidase on Avicel glucan, on birchwood xylan, and on corn stover pretreated with ammonia fiber expansion (AFEX) and dilute acid over a protein loading range from 0 to 15 mg/ml.

Cellulase and hemicellulase adsorption on Avicel, birchwood xylan and solids from AFEX and dilute acid pretreatment of corn stover were measured to determine the interaction of different enzymes with these substrates. Figure 4 shows that the adsorption data followed the Langmuir relationship well over the range of protein loadings of 0-15 mg/ml used and the adsorption parameters of the enzymes on these substrates estimated by non-linear regression of adsorption data to the Langmuir equation are given in Table 2. Spezyme CP cellulase had a higher protein-adsorption capacity and affinity for birchwood xylan than for Avicel glucan, consistent with the finding by Kanda and coworkers that endo-glucanase binds to xylan > to cellulose and has an even greater Michaelis constant (*K*_*m*_) for xylan than for cellulose [35]. This result could be due to the rigid crystalline structure of Avicel glucan limiting the surface available for enzyme adsorption compared with the looser amorphous structure of xylan. However, this observation could also be explained by xylan or its derivatives competitively inhibiting Spezyme CP cellulase. In addition, the higher xylan content of AFEX-pretreated corn stover could result in stronger protein adsorptions than on dilute acid-pretreated corn stover due to relatively higher adsorption affinities of cellulase on xylan and lignin than cellulose. However, in one of our previous studies, it was found that AFEX lignin had lower affinity to xylanase than dilute acid lignin, although dilute acid corn stover used in that study was prepared at different conditions and using a different kind of reactor [24]. On the other hand, Novozyme 188 β-glucosidase had a much lower adsorption capacity and affinity for all of these substrates than cellulase. In fact, β-glucosidase adsorption parameters were higher for pretreated corn stover than for pure cellulose or xylan, probably due to its greater affinity for lignin shown in previous research [24,36].

**Table 2:** Maximum protein-adsorption capacity, affinity constants and correlation coefficients of different enzymes with Avicel, birchwood xylan and solids from corn stover (CS) pretreated with dilute acid and ammonia fiber expansion (AFEX).

Enzyme	Substrates	**σ, mg/g substrate**^**1**^	**Kd, Liter/g**^**2**^	***R***^**2**^
Spezyme CP cellulase	Avicel	82.4	1.5	0.98
	
	Birchwood xylan	99.7	2.5	0.97
	
	AFEX CS	102.7	2.7	0.94
	
	Dilute acid CS	92.7	2.8	0.98

Novozyme 188 β-glucosidase	Avicel	52.8	1.3	0.87
	
	Birchwood xylan	46.3	1.1	0.82
	
	AFEX CS	78.7	1.8	0.91
	
	Dilute acid CS	73.6	1.7	0.95

Multifect xylanase	Avicel	60.8	1.9	0.94
	
	Birchwood xylan	114.1	2.7	0.96
	
	AFEX CS	85.7	1.8	0.87
	
	Dilute acid CS	74.3	1.9	0.92

Genencor	Avicel	82.3	2.6	0.95
	
β-xylosidase	Birchwood xylan	115.2	3.7	0.91
	
	AFEX CS	98.1	2.3	0.97
	
	Dilute acid CS	88.5	2.4	0.89

^1^Maximum cellulase adsorption capacity.

^2^Affinity constant.

The two hemicellulase preparations had a much stronger adsorption preference for birchwood xylan than Avicel glucan. For Multifect xylanase on birchwood xylan, the adsorption capacity was 114.1 mg/g and the affinity was 2.7 L/g, compared with just 60.8 mg/g adsorption capacity and 1.9 L/g adsorption affinity for Avicel cellulose. Similarly, β-xylosidase found stronger binding to birchwood xylan than Avicel cellulose but had even higher adsorption parameters than xylanase. In addition, β-xylosidase adsorption was stronger on AFEX-pretreated corn-stover solids than on dilute acid-pretreated corn-stover solids, possibly as a result of the higher xylan content of the AFEX processed material. Overall, both hemicellulase activities displayed greater adsorption on higher xylan content substrates whereas higher glucan content did not necessarily result in stronger cellulase binding. These observations suggest that cellulase competitively binds to xylan and thereby could provide a possible mechanism for xylan or xylo-oligomer inhibition [5,18,37,38]. However, the hypothesis of protein adsorption to soluble/insoluble xylo-oligomers is still to be proved.

### The effects of enzyme-supplementation sequence

Although the mechanism is still subject to debate, cellulose digestion generally improves with xylan removal by chemical or enzymatic treatment, and it is widely believed that both lignin and hemicelluloses form a physical barrier that hinders access by cellulase [33,39]. In addition, enzymatic removal of xylan and glucomannan were shown to enlarge the pore size of pine or birch Kraft pulps [40]. Thus, studies of this nature attribute the key benefit of xylan removal to enhancement of substrate accessibility to enzymes through exposing more crystalline cellulose surface. However, because our recent research suggested that soluble xylo-oligomers released from xylan during enzymatic hydrolysis could be powerful inhibitors of cellulase activity, removing xylan before adding cellulase could therefore improve enzyme effectiveness. The protein-adsorption data of this study, which show a stronger binding of cellulase to birchwood xylan than to Avicel cellulose, and the possibility of competitive inhibition of cellulase by xylan and xylan derivatives, are consistent with the latter reasoning.

**Figure 5:**
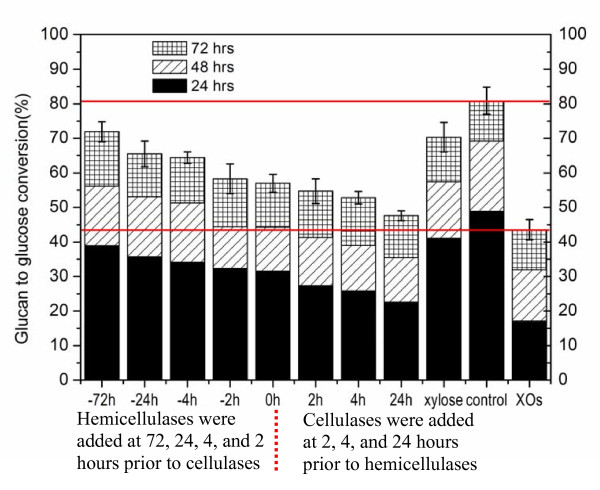
**Conversion of glucan in Avicel to glucose with change in time for adding hemicellulase before (or after) cellulase addition for enzymatic hydrolysis at 50°C and pH 4.8 at an enzyme loading of 5 **filter paper units (FPU))**/g glucan and 10 cellobiase units (CBU)/g glucan with 2% Avicel glucan**. Multifect xylanase and β-xylosidase were supplemented at a loading of 30 mg protein/g equivalent xylose (xylanase: β-xylosidase 2:1). control refers to Avicel (without xylo-oligomers) with an enzyme loading of 5 FPU and 10 CBU/g glucan but no added hemicellulases. The xylo-oligomer control (XOs) was a 2% Avicel loading sample supplemented with 12.5 mg/ml (equivalent xylose concentration) mixed degree of polymerization xylo-oligomers hydrolyzed by cellulase and β-glucosidase at the same dosage as for the control. The xylose sample refers to Avicel hydrolysis with 12.5 mg/ml pure xylose at the same enzyme loadings for comparison.

Based on these observations, we hypothesized that removing xylan and xylo-oligomers or hydrolyzing them to xylose before adding cellulase would enhance cellulase efficacy. In addition, the cellulase and hemicellulase adsorption data suggest that adding the different enzymes in multiple steps would prevent undesirable binding. Therefore, xylanase and β-xylosidase were added before and after cellulase addition, to evaluate their effects on inhibition under different scenarios. There was a marked trend (Figure 5) indicating that applying xylanase and β-xylosidase a few hours before adding cellulase significantly improves cellulose conversion. For example, if xylanase and β-xylosidase were added 24 hours before cellulase (sample marked as -24 h), the conversion after 72 hours was 65.5%, compared with just 49.4% when these two hemicellulases were added 24 hours after cellulase (sample marked as 24 h). Thus, the greatest alleviation of inhibition occurred when the xylo-oligomers were hydrolyzed to xylose after 72 hours. By contrast, when xylanase and β-xylosidase were added after cellulase, their effects weakened considerably. In light of the adsorption data (Table 2), this observation could be attributed to competitive adsorption of cellulase and hemicellulases to cellulose and xylo-oligomers. Thus, when hemicellulases were added before cellulase, hemicellulases' higher binding affinity would result in greater binding to xylan or xylo-oligomers, and reduce the opportunity for the cellulase that was added later to bind to hemicelluloses. From this perspective, enzyme efficacy could not only be improved by modifying the cocktail composition so as to increase xylanase activity, but also by rational strategies for enzyme addition that avoid unproductive losses.

### Implications for possible mechanisms

The key findings of this research suggest competitive binding of cellulase and hemicellulase on cellulose reactive sites (Figure 1), and as a result, the efficacy of cellulase is reduced by comparable amounts of hemicellulase protein. However, if the substrate has relatively high xylan content, hemicellulases preferably bind to hemicelluloses and also hydrolyze inhibitory xylo-oligomers to enhance the overall glucose and xylose yields (Figure 2, Figure 3). In addition, Multifect xylanase and Genencor β-xylosidase have a higher binding affinity to xylan than to glucan, thereby reducing the possibility of steric hindrance of one by the other when used with substrates of high xylan content. By contrast, cellulase binds more strongly to xylan than glucan, whereas β-glucosidase binds primarily to higher lignin content substrates (Table 2). Therefore, xylan, xylo-oligomers and lignin seem to reduce cellulase availability to react with cellulose by undesirable binding with these proteins. As supported by other research, supplementation with xylanase and β-xylosdiase could reduce this negative effect by hydrolyzing xylan and its oligomers to xylose (unpublished data). IN addition, this research further indicated that adding hemicellulase before cellulase could enhance the enzymatic benefits by reducing competitive binding of cellulases to xylan and xylo-oligomers instead of to cellulose. These findings highlight the need to modify enzyme-cocktail compositions and to have rational strategies for advancing enzyme efficacy.

## Conclusions

Adding xylanase or β-xylosidase improved the enzymatic hydrolysis of cellulose and hemicellulose in solids after AFEX and dilute acid pretreatment of corn stover. Xylan removal has been widely believed to disrupt barriers that hinder enzyme access to cellulose, but this research suggests that cellulase binding to xylan and, possibly, xylo-oligomers strongly reduces cellulase activity, and that xylan and xylo-oligomer removal or conversion to xylose can greatly reduce their inhibition. Chemically or enzymatically removing xylan and xylo-oligomers before adding cellulase seems to provide a particularly important method of enhancing cellulase effectiveness and thereby reducing the doses needed to achieve a given performance.

## Competing interests

The authors declare that they have no competing interests.

## Authors' contributions

QQ designed and carried out experiments, analyzed the results and wrote the manuscript. CEW analyzed the results and reviewed the manuscript. All authors read and approved the final manuscript.

## References

[B1] Farrell AE Ethanol can contribute to energy and environmental goals (vol 311, pg 506, 2006) Science 2006 312 1748 1748 16439656 10.1126/science.1121416

[B2] Wyman CE Dale BE Elander RT Holtzapple M Ladisch MR Lee YY Comparative sugar recovery data from laboratory scale application of leading pretreatment technologies to corn stover Bioresource Technol 2005 96 2026 2032 10.1016/j.biortech.2005.01.018 16112491

[B3] Lynd LR Laser MS Brandsby D Dale BE Davison B Hamilton R Himmel M Keller M McMillan JD Sheehan J Wyman CE How biotech can transform biofuels Nature Biotechnology 2008 26 169 172 10.1038/nbt0208-169 18259168

[B4] Himmel ME Ruth MF Wyman CE Cellulase for commodity products from cellulosic biomass Current Opinion in Biotechnology 1999 10 358 364 10.1016/S0958-1669(99)80065-2 10449322

[B5] Gao DH Chundawat SPS Krishnan C Balan V Dale BE Mixture optimization of six core glycosyl hydrolases for maximizing saccharification of ammonia fiber expansion (AFEX) pretreated corn stover Bioresource Technol 2010 101 2770 2781 10.1016/j.biortech.2009.10.056 19948399

[B6] Bayatian GL Chatrchyan S Hmayakyan G Sirunyan AM Adam W Bergauer T Dragicevic M Ero J Friedl M Fruehwirth R CMS physics technical design report, volume II: Physics performance Journal of Physics G-Nuclear and Particle Physics 2007 34 995 1579 10.1088/0954-3899/34/6/S01

[B7] Xiao ZZ Zhang X Gregg DJ Saddler JN Effects of sugar inhibition on cellulases and beta-glucosidase during enzymatic hydrolysis of softwood substrates Appl Biochem Biotech 2004 113-16 1115 1126 10.1385/abab:115:1-3:1115 15054257

[B8] Selig MJ Knoshaug EP Adney WS Himmel ME Decker SR Synergistic enhancement of cellobiohydrolase performance on pretreated corn stover by addition of xylanase and esterase activities Bioresource Technol 2008 99 4997 5005 10.1016/j.biortech.2007.09.064 18006303

[B9] Chen HC Grethlein HE Effect of cellulase size-reduction on activity and accessibility Biotechnol Lett 1988 10 913 918 10.1007/BF01027005

[B10] Jeoh T Ishizawa CI Davis MF Himmel ME Adney WS Johnson DK Cellulase digestibility of pretreated biomass is limited by cellulose accessibility Biotechnol Bioeng 2007 98 112 122 10.1002/bit.21408 17335064

[B11] Saddler JN Mooney CA Mansfield SD Beatson RP Influence of fiber characteristics on the cellulase accessibility to softwoods Abstr Pap Am Chem S 1999 217 U264 U265

[B12] Kumar R Wyman CE Does change in accessibility with conversion depend on both the substrate and pretreatment technology? Bioresource Technol 2009 100 4193 4202 10.1016/j.biortech.2008.11.058 19398329

[B13] Converse AO Matsuno R Tanaka M Taniguchi M A model of enzyme adsorption and hydrolysis of microcrystalline cellulose with slow deactivation of the adsorbed enzyme Biotechnol Bioeng 1988 32 38 45 10.1002/bit.260320107 18584716

[B14] Eriksson T Karlsson J Tjerneld F A model explaining declining rate in hydrolysis of lignocellulose substrates with cellobiohydrolase I (Cel7A) and endoglucanase I (Cel7B) of *Trichoderma reesei* Applied Biochemistry and Biotechnology 2002 101 41 60 10.1385/ABAB:101:1:41 12008866

[B15] Holtzapple M Cognata M Shu Y Hendrickson C Inhibition of *Trichoderma-Reesei *cellulase by sugars and solvents Biotechnology and Bioengineering 1990 36 275 287 10.1002/bit.260360310 18595079

[B16] Scheiding W Thoma M Ross A Schugerl K Modeling of the enzymatic-hydrolysis of cellobiose and cellulose by a complex enzyme mixture of *Trichoderma-Reesei Qm 9414* Applied Microbiology and Biotechnology 1984 20 176 182

[B17] Ghose TK Bisaria VS Studies on the mechanism of enzymatic-hydrolysis of cellulosic substances Biotechnol Bioeng 1979 21 131 146 10.1002/bit.260210110 106903

[B18] Qing Q Yang B Wyman CE Xylooligomers are strong inhibitors of cellulose hydrolysis by enzymes Bioresource Technol 2010 101 9624 9630 10.1016/j.biortech.2010.06.137 20708404

[B19] Sluiter A Hames B Ruiz R Scarlata C Sluiter J Templeton D Crocker D Determination of structural carbohydrates and lignin in biomass Technique report, NREL/TP-510-42618 2008

[B20] Lloyd TA Wyman CE Combined sugar yields for dilute sulfuric acid pretreatment of corn stover followed by enzymatic hydrolysis of the remaining solids Bioresource Technology 2005 96 1967 1977 10.1016/j.biortech.2005.01.011 16112484

[B21] Gray MC Converse AO Wyman CE Solubilities of oligomer mixtures produced by the hydrolysis of xylans and corn stover in water at 180 degrees C Industrial & Engineering Chemistry Research 2007 46 2383 2391 10.1021/ie060325+21706774

[B22] Selig M Weiss NYJ Enzymatic saccharification of lignocellulosic biomass Technique report, NREL/TP-510-42629 2008

[B23] Kumar R Wyman CE An improved method to directly estimate cellulase adsorption on biomass solids Enzyme Microb Tech 2008 42 426 433 10.1016/j.enzmictec.2007.12.005

[B24] Kumar R Wyman CE Cellulase adsorption and relationship to features of corn stover solids produced by leading pretreatments Biotechnology and Bioengineering 2009 103 252 267 10.1002/bit.22258 19195015

[B25] Lynd LR Weimer PJ van Zyl WH Pretorius IS Microbial cellulose utilization: Fundamentals and biotechnology Microbiol Mol Biol R 2002 66 506 + 10.1128/MMBR.66.3.506-577.2002 PMC120791 12209002

[B26] Mosier N Wyman C Dale B Elander R Lee YY Holtzapple M Ladisch M Features of promising technologies for pretreatment of lignocellulosic biomass Bioresource Technol 2005 96 673 686 10.1016/j.biortech.2004.06.025 15588770

[B27] Wyman CE Dale BE Elander RT Holtzapple M Ladisch MR Lee YY Coordinated development of leading biomass pretreatment technologies Bioresource Technol 2005 96 1959 1966 10.1016/j.biortech.2005.01.010 16112483

[B28] Teymouri F Laureano-Perez L Alizadeh H Dale BE Ammonia fiber explosion treatment of corn stover Appl Biochem Biotech 2004 113-16 951 963 10.1385/abab:115:1-3:0951 15054244

[B29] Teymouri F Laureano-Perez L Alizadeh H Dale BE Optimization of the ammonia fiber explosion (AFEX) treatment parameters for enzymatic hydrolysis of corn stover Bioresource Technol 2005 96 2014 2018 10.1016/j.biortech.2005.01.016 16112489

[B30] Boussaid AL Esteghlalian AR Gregg DJ Lee KH Saddler JN Steam pretreatment of Douglas-fir wood chips - Can conditions for optimum hemicellulose recovery still provide adequate access for efficient enzymatic hydrolysis? Appl Biochem Biotech 2000 84-6 693 705 10.1385/abab:84-86:1-9:693 10849828

[B31] Fernandez-Bolanos J Felizon B Heredia A Rodriguez R Guillen R Jimenez A Steam-explosion of olive stones: hemicellulose solubilization and enhancement of enzymatic hydrolysis of cellulose Bioresource Technol 2001 79 53 61 10.1016/S0960-8524(01)00015-3 11396908

[B32] Mussatto SI Fernandes M Milagres AMF Roberto IC Effect of hemicellulose and lignin on enzymatic hydrolysis of cellulose from brewer's spent grain Enzyme Microb Tech 2008 43 124 129 10.1016/j.enzmictec.2007.11.006

[B33] Ohgren K Bura R Saddler J Zacchi G Effect of hemicellulose and lignin removal on enzymatic hydrolysis of steam pretreated corn stover Bioresource Technol 2007 98 2503 2510 10.1016/j.biortech.2006.09.003 17113771

[B34] Yoshida M Liu Y Uchida S Kawarada K Ukagami Y Ichinose H Kaneko S Fukuda K Effects of cellulose crystallinity, hemicellulose, and lignin on the enzymatic hydrolysis of *Miscanthus sinensis *to monosaccharides Bioscience Biotechnology and Biochemistry 2008 72 805 810 10.1271/bbb.70689 18323635

[B35] Kanda T Wakabayashi K Nisizawa K Xylanase activity of an endo-cellulase of carboxymethyl-cellulase type from Irpex-Lacteus (Polyporus-Tulipiferae) J Biochem-Tokyo 1976 79 989 995 8438 10.1093/oxfordjournals.jbchem.a131166

[B36] Yang B Wyman CE BSA treatment to enhance enzymatic hydrolysis of cellulose in lignin containing substrates Biotechnology and Bioengineering 2006 94 611 617 10.1002/bit.20750 16673419

[B37] Kumar R Wyman CE Effect of xylanase supplementation of cellulase on digestion of corn stover solids prepared by leading pretreatment technologies Bioresource Technol 2009 100 4203 4213 10.1016/j.biortech.2008.11.057 19386492

[B38] Meshartree M Saddler JN The nature of inhibitory materials present in pretreated lignocellulosic substrates which inhibit the enzymatic-hydrolysis of cellulose Biotechnol Lett 1983 5 531 536 10.1007/BF01184944

[B39] Yang B Wyman CE Effect of xylan and lignin removal by batch and flowthrough pretreatment on the enzymatic digestibility of corn stover cellulose Biotechnology and Bioengineering 2004 86 88 95 10.1002/bit.20043 15007845

[B40] Suurnakki A Li TQ Buchert J Tenkanen M Viikari L Vuorinen T Odberg L Effects of enzymatic removal of xylan and glucomannan on the pore size distribution of kraft fibres Holzforschung 1997 51 27 33 10.1515/hfsg.1997.51.1.27

